# Validity and reliability of SEIS-3: An instrument for subjective measuring of strength in older adults

**DOI:** 10.1016/j.mex.2023.102512

**Published:** 2023-12-04

**Authors:** Renato Sobral-Monteiro-Junior, Luciana Aparecida Coutinho, Vinícius Dias Rodrigues, Frederico Sander Mansur Machado, Wellington Danilo Soares, Henrique Nunes Pereira Oliva, Camila Cristina Fonseca Bicalho

**Affiliations:** a Study and Research Group in Neuroscience, Exercise, Health and Sport (GENESEs), Physical Education Department, State University of Montes Claros, Montes Claros, Minas Gerais, Brazil; b Physical Education Department, State University of Montes Claros, Montes Claros, Minas Gerais, Brazil; c Department of Psychiatry, Yale University School of Medicine, New Haven, CT, USA; d Human Movement Sciences Department, State University of Minas Gerais, Ibirité, Minas Gerais, Brazil; e University of Religions and Denominations, Qom, Iran

**Keywords:** Physical exertion, Elderly, Strength training, Resistance training, Subjective Effort Induction Scale (SEIS-3)

## Abstract

The use of rating of perceived exertion (RPE) has grown substantially, providing a valuable alternative for exercise intensity monitoring, especially for older adults. However, some challenges, such as cross-cultural validity, age-related differences, and reliability issues, necessitate the development of a concise and user-friendly RPE instrument, particularly for strength training in this population. This study aimed to validate the Subjective Effort Induction Scale (SEIS-3), a simplified tool for measuring effort during strength training. SEIS-3 is a graded instrument with three exertion levels: 1) Light effort, 2) Moderate effort, and 3) Strong effort. Twenty seniors, aged 71±7 years, of both genders participated in the study (CEP/Unimontes 2,741,071/2018). We collected maximal isometric voluntary contraction (MIVC) data using a digital dynamometer. Subsequently, participants underwent the initial test, following the three SEIS-3 categories in a randomly assigned order of induced subjective effort. SEIS-3 instrument was evaluated by the approaches:

• Linear regression analysis: employed to assess the validity of the instrument.
• Intraclass Correlation Coefficient (ICC): employed to assess the reliability of the instrument.

SEIS-3 effort categories correlated with MIVC in both hands (R^2^=0.80, *F* = 25.596, df=3, *p* < 0.01 for right hand; R^2^=0.56, *F* = 9.132, *p* < 0.01 for left hand). Test-retest reliability for grip strength across effort categories was excellent (ICC > 0.9). SEIS-3 is a valid and reliable user-friendly tool for accurately assessing and regulating exercise intensity in older adults during strength tasks, benefiting their health, functional capacity, and overall quality of life. This low-cost instrument can help health professionals in their activities.

## Graphical abstract

**Figure:**
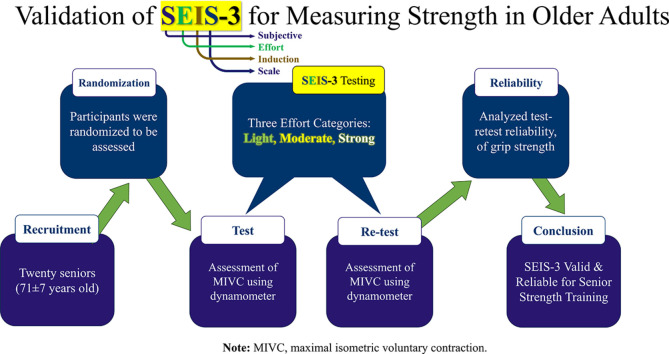

Specifications table

**Table:** 

Subject area:	Medicine and Dentistry
More specific subject area:	Resistance Training
Name of your method:	Subjective Effort Induction Scale (SEIS-3)
Name and reference of original method:	N/A
Resource availability:	N/A

## Method details

### Context and significance

Rating of perceived exertion (RPE) has been widely used for more than 50 years to measure the intensity of exercise [1], [2], [3]. RPE is a simple and easy-to-use instrument to monitor a person's effort during different types of exercise, such as aerobic and strength training [1,4]. The Borg Scale, one of the most renowned RPE scales, was developed in 1970 and demonstrates a significant association with somatic stress and internal load during exercise [2,5]. Since its creation, the Borg Scale and its subsequent updates have been extensively employed in diverse populations [4,[6], [7], [8]. Another commonly used subjective perceived exertion scale is the Omni Scale, which was developed in 2003 to assess RPE in resistance training [9]. Borg and Omni scales have been applied in research and practical settings of exercise training.

The use of RPE is growing as it could replace traditional methods of intensity monitoring. The elderly population has unique vital parameters and exercise requirements that warrants specific evaluation, as highlighted by recent research focused on this demographic [10,11]. For instance, Row et al. [12] investigated whether RPE would predict different percentages of explosive resistance training in healthy older adults. They found a strong correlation between the average RPE and the percentage of 1RM (R^2^ = 99.5 %; *p* < 0.001), suggesting that RPE could be used to simplify the intensity prescription of explosive resistance training for older adults instead of traditional approaches (%1RM). Furthermore, a review carried out by Morishita et al. [4] shows evidence of the application of RPE in resistance training. However, the authors mention the difficulty of proving the agreement of RPE and%1RM.

While RPE is useful for monitoring the training intensity of elite athletes [13], some concerns have been identified. Cabral et al. [1] suggested that the language of the Borg Scale should be cross-culturally validated, to prevent misunderstandings resulting from simple translations. Moreover, age seems to be a relevant influence on the RPE since children and adults perceive different efforts differently [14]. Additionally, sedentary older adults seem to misclassify their exertion during a maximal cycling test compared to young adults [15]. These findings indicate the importance of caution when using RPE with older adults. Although RPE generally exhibits acceptable reliability, the level of agreement varies from moderate to high and sometimes substantial, particularly among older adults [16,17]. The number of effort levels in RPE scales may impact the comprehension of older adults, making it challenging for them to accurately perceive exertion and regulate their effort levels [14,15]. Misclassification of the effort may occur because of a high cognitive demand to correlate the physiological load and the interpretation of visual or auditory information regard the intensity. Central and peripheral inputs isolated or in conjunction may vary the effort perception [3,18]. Considering these factors, there is a need to develop a new RPE instrument, easily understandable, that can effectively monitor and prescribe exercise intensity for this population, especially regarding strength. A more concise and shorter RPE instrument could minimize bias in the subjective measurement of strength.

The aim of this study was to develop and validate a subjective effort induction scale (SEIS) specifically designed for older adults performing strength exercise. It was hypothesized that a simple 3-point scale would be valid for inducing and accurately representing different levels of strength exertion. Additionally, it was hypothesized that the scale would exhibit acceptable reliability when used to assess strength tasks at different time points.

### Study design

This study employed a cross-sectional design at two time points to validate a new instrument for inducing and measuring different degrees of effort in older adults.

### Sample

The sample size was determined using Clincalc, a statistical calculator (https://clincalc.com/stats/samplesize.aspx). The following parameters were considered: (i) Study group design: one group versus population, ii) Primary end point: continuous; (iii) Statistical parameters: mean (25.79 kgf) and standard deviation (9.71 kgf) of handgrip strength from the population, mean (19.95 kgf) of handgrip strength from a preliminary sample of 15 subjects from our laboratory; iv) Alpha value: 0.05; and v) Power: 80 %.

Data from the population were obtained from a previous study [19] that included data from older adults in Minas Gerais, a Brazilian region where the current data collection took place. The sample size calculation determined that a total of 20 individuals would be necessary for this study.

### Participants

Participants aged 60 years or older, who could walk independently and exhibited cognitive health, were included in the study. Individuals with severe cardiovascular disease, musculoskeletal injury, diabetes, physical restrictions, cognitive impairment or cerebrovascular disease were excluded from the study. A total of 20 individuals voluntarily provided an informed consent and agreed to participate in the study, which received approval from the local ethics commission (State University of Montes Claros - protocol 2.741.071/2018).

### Procedures

A 3-point Subjective Effort Induction Scale (SEIS-3) was developed, consisting of three scores: (1) Light effort, (2) Moderate effort, and (3) Strong effort. SEIS-3 is depicted in Box I (Supplementary Material). Participants from a project carried out in the Exercise Laboratory of the State University of Montes Claros were instructed to hold a digital dynamometer (DM-90, Itest, Bosque da Saúde, São Paulo, Brazil) and perform a grip strength test, applying maximal isometric voluntary contraction (MIVC) for five seconds with both hands. A familiarization procedure was carried out before the test. Subsequently, they completed the initial assessment of the three categories of SEIS-3 in a randomly assigned order of induced subjective effort. The same procedure was repeated after a 10-minute rest period (re-test). The strength (kgf) reached in each category of SEIS-3 was divided by the data from MIVC (kgf) to calculate a percentage of MIVC.

### Data analysis

Firstly, we identified how much each category of SEIS-3 would comprise the MIVC. For this reason, a percentage of MIVC was calculated based on the effort performed (i.e., weak effort = low percentage of MIVC; moderate effort = medium percentage of MIVC; strong effort = high percentage of MIVC).

To analyze the evidence of validity for SEIS-3, a linear regression analysis was conducted to examine the relationship between the strength reached in each category of the scale and the MIVC data, assessing whether SEIS-3 could explain the variance in MIVC (R^2^). The Intraclass Correlation Coefficient (ICC) was calculated to assess the test-retest reliability (R). Additionally, Bland-Altman plots were used to analyze the 95 % of agreement of the measurement in all different time points. JASP software [20] and VassarStats [21] were used to handle and analyze the data.

### Sample characteristics

A total of 20 individuals (14 females) were recruited for the study. The participants had a mean age of 71 ± 7 years, mean weight of 69 ± 13 kg, and mean height of 161 ± 9 cm.

## Method validation

### Validity and reliability of SEIS-3

The strength levels achieved in all effort categories of SEIS-3 exhibited a proportional relationship with maximal isometric voluntary contraction (MIVC) in both the right hand (R^2^ = 0.80, *F* = 25.596, df = 3, *p* < 0.01) and the left hand (R^2^ = 0.56, *F* = 9.132, *p* < 0.01). Each effort category was independently proportional to total MIVC in both hands, as follows: Right hand - Weak effort (R^2^ = 0.18, *F* = 5.171, df = 1, *p* < 0.01); Moderate effort (R^2^ = 0.21, *F* = 5.999, df = 1, *p* = 0.02); Strong effort (R^2^ = 0.35, *F* = 11.124, df = 1, *p* < 0.01); Left hand - Weak effort (R^2^ = 0.13, *F* = 3.743, df = 1, *p* = 0.07); Moderate effort (R^2^ = 0.21, *F* = 6.059, df = 1, *p* = 0.02); Strong effort (R^2^ = 0.27, *F* = 8.191, df = 1, *p* = 0.01). Figure I in the Supplementary Material illustrates the relationship between the strength reached in each SEIS-3 category and MIVC for both the right and left hands Each category of SEIS-3 comprised three different percentages of MIVC, as expected <60 %, >60 %, >80 % (Figure II in Supplementary Material). The right hand demonstrated higher validity compared to the left hand. The assumptions for regression were met. The autocorrelation (Autocorrelation = 0.0693, Durbin-Watson = 1.75, *p* = 0.63) and collinearity statistics (mean weak effort VIF = 1.09, mean moderate effort VIF = 1.20, mean strong effort VIF = 1.13) were not significant.

**Fig. 1:**
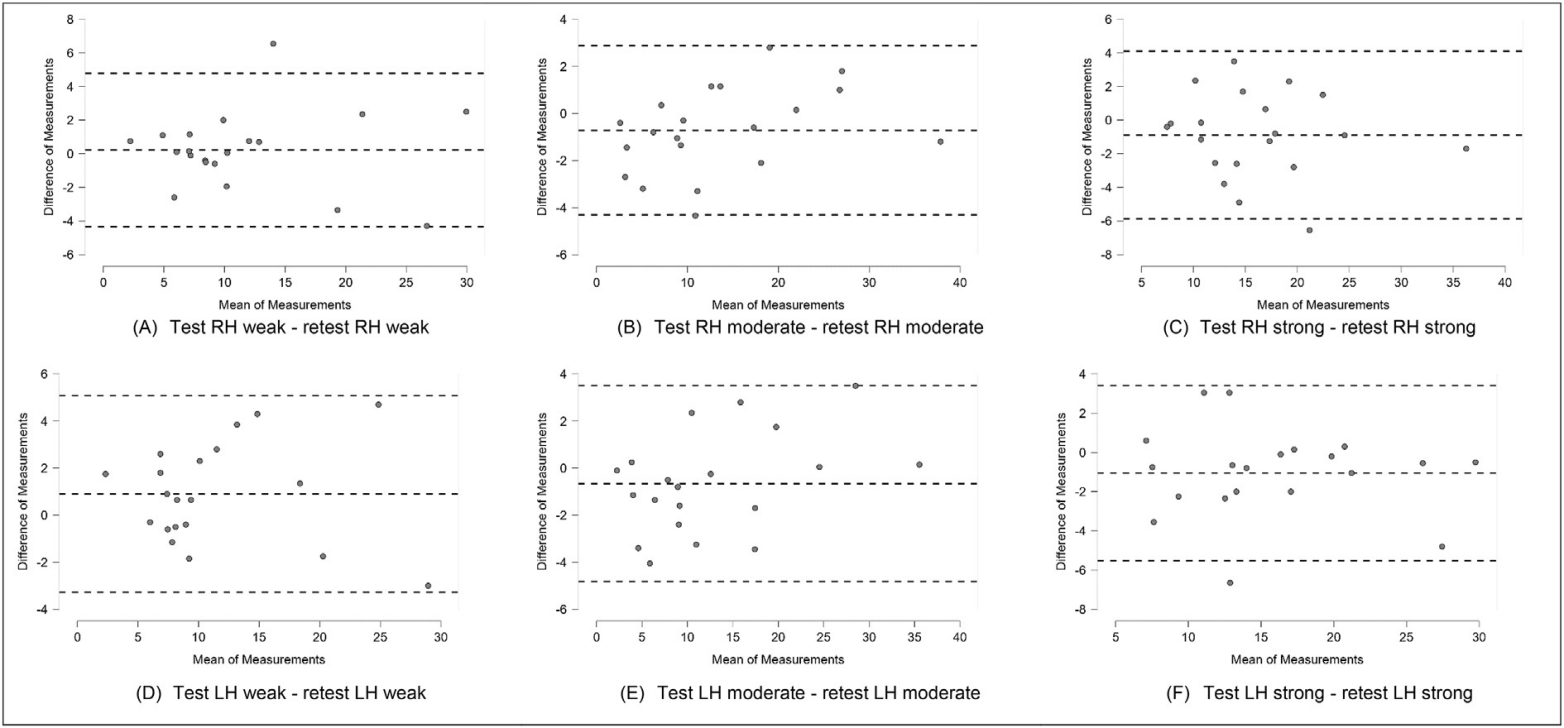
Bland-Altman Plots: (A) right hand weak effort, (B) right hand moderate effort, (C) right hand strong effort, (D) left hand weak effort, (E) left hand moderate effort, (F) left hand strong effort. Note: RH, right hand; LH, left hand.

The reproducibility, or test-retest reliability, of grip strength obtained through each effort category with both hands at different times was considered excellent, with an Intraclass Correlation Coefficient (R) greater than 0.9 (Table 1). Bland-Altman plots display the excellent agreement between measures in two time points of both hands (Fig. 1).

**Table 1:** Intraclass Coefficient Correlation of SEIS-3, with each category of effort (weak, moderate, and strong) in different applications (test vs. re-test).

Hands, effort	Test_kgf_ (mean ± SD)	*Re*-test_kgf_ (mean ± SD)	R (F)	p-value
Right hand, SEIS-3 weak effort	11.7 ± 7.3	11.54 ± 7.43	0.95 (41.2)	<0.01
Right hand, SEIS-3 moderate effort	13.2 ± 9.7	13.9 ± 9	0.97 (93.4)	<0.01
Right hand, SEIS-3 strong effort	15.7 ± 6.6	16.6 ± 6.9	0.92 (25.7)	<0.01
Left hand, SEIS-3 weak effort	11.9 ± 6.8	11 ± 6.8	0.94 (35.4)	<0.01
Left hand, SEIS-3 moderate effort	12.4 ± 9.3	13 ± 8.5	0.97 (66.8)	<0.01
Left hand, SEIS-3 strong effort	15.3 ± 6.6	16.3 ± 6.7	0.93 (28.9)	<0.01

Degree of freedom is 19 in all analyses; SD: standard deviation; kgf: kilogram-force.

### Study implications

This study aimed to present evidence of validity and verify the reliability of an easy scale designed to subjectively induce different graded efforts in older adults during strength training. The SEIS-3 instrument achieved 80 % of the MIVC component in the dominant hand and demonstrated excellent reproducibility, reaching high agreement (95 %) in the Bland-Altman limits. Additionally, different effort categories were aligned with different percentages of MIVC, with low and high efforts corresponding to low and high percentages of maximal contraction, respectively.

RPE have been widely used to monitor perceive9d effort during exercise for many years [2]. However, these instruments are usually applied to monitor the effort made during exercise. The most widely recognized instruments in the literature for measuring RPE are the Borg Scale (and its versions) and the OMNI Scale [1,^2^,9]. Nevertheless, both instruments have 10 or more effort categories, which could make it difficult for older adults and other populations to truly understand and express the intensity of exercise, leading to misinterpretation of self-regulation [8,^15^,22]. In this context, the evidence of validity of the instrument in this present study demonstrates an interesting application. Firstly, it was designed to induce and measure effort during a strength task. Secondly, SEIS-3 effectively induces effort in three different ranges of%MIVC (<60 %, >60 %, >80 %; Figure II in Supplementary Material). Finally, the scale is understandable and easily applicable for older adults, as they can interpret a few categories of effort, minimizing bias regarding high cognitive demand and misunderstandings of self-regulation.

Despite the promising findings presented here, some limitations must be outlined. While the handgrip dynamometer used in this research is valuable for quantitatively measuring isometric strength, the results may vary when applying SEIS-3 to exercises primarily involving isotonic contractions. Therefore, it is recommended to verify the effort measured by SEIS-3 through multiple attempts during traditional strength training using machines and free weights. In the strength training program for older adults using isotonic muscle contractions, it is advised to familiarize oneself with the load in each exercise and then apply SEIS-3. Finally, the measure of strength in the present study was performed using upper limbs, but not lower limbs. Despite these limitations, the validity, reliability, easy design, and clarity of SEIS-3 establish it as a feasible instrument to be implemented in practical applications.

Future research directions stemming from the study on SEIS-3 could encompass clinical applications in diverse patient populations, integration with technology for real-time tracking, longitudinal assessment of long-term benefits, training programs for effective implementation, comparisons with other intensity-monitoring tools, exploration of psychosocial factors impacting adherence and outcomes. These avenues of investigation hold the potential to broaden the SEIS-3′s scope in advancing its utility in strength training and exercise programs.

In conclusion, as a strength of the present work, SEIS-3 was found to be a valid and reliable instrument for inducing and measuring effort in older adults during strength task. Simple design and ease of use of our instrument make it a valuable tool for accurately assessing and regulating exercise intensity in this population. The application of SEIS-3 extends to various practical contexts, including exercise prescription and monitoring, research investigations into the physiological responses to effort, and personalized interventions in clinical and rehabilitation settings. By facilitating accurate assessment and regulation of exercise intensity, SEIS-3 contributes to promoting safe and effective strength training interventions for older adults, enhancing their overall health, functional capacity, and quality of life. This low-cost instrument can help researchers and health professionals in the research settings and clinical practice, respectively.

## Ethics statements

Participants signed a consent form to participate in this study, which was approved by a local ethics committee (Protocol 2.741.071/2018).

## Declaration of competing interest

The authors declare that they have no known competing financial interests or personal relationships that could have appeared to influence the work reported in this paper.

## CRediT authorship contribution statement

**Renato Sobral-Monteiro-Junior:** Conceptualization, Methodology, Software, Funding acquisition, Writing – original draft, Writing – review & editing. **Luciana Aparecida Coutinho:** Validation, Data curation, Writing – original draft. **Vinícius Dias Rodrigues:** Writing – review & editing, Visualization, Investigation. **Frederico Sander Mansur Machado:** Writing – review & editing. **Wellington Danilo Soares:** Data curation, Software, Validation. **Henrique Nunes Pereira Oliva:** Writing – review & editing. **Camila Cristina Fonseca Bicalho:** Conceptualization, Methodology, Writing – original draft, Writing – review & editing.

## Data Availability

Data will be made available on request.

## Appendix Supplementary materials

Image, application 1
