# Supplementary material for: Validity and reliability of SEIS-3: An instrument for subjective measuring of strength in older adults
Source: MethodsX. 2023 Dec 4;12:102512. doi: 10.1016/j.mex.2023.102512 (PMC10750096; doi:10.1016/j.mex.2023.102512)

**SUPPLEMENTARY MATERIAL**

**Article title:** Validity and reliability of SEIS-3: an instrument for subjective measuring of strength in older adults.

**Author names and affiliations:** Renato Sobral Monteiro-Junior^1^, Luciana Aparecida Coutinho^1^, Vinícius Dias Rodrigues^1^, Frederico Sander Mansur Machado^1^, Wellington Danilo Soares^2^, Henrique Nunes Pereira Oliva^3^, Camila Cristina Fonseca Bicalho^4^

^1^ Study and Research Group in Neuroscience, Exercise, Health and Sport (GENESEs), Physical Education Department, State University of Montes Claros, Montes Claros, Minas Gerais, Brazil

^2^ Physical Education Department, State University of Montes Claros, Montes Claros, Minas Gerais, Brazil

^3^ Department of Psychiatry, Yale University School of Medicine, New Haven, CT, USA

^4^ Human Movement Sciences Department, State University of Minas Gerais, Ibirité, Minas Gerais, Brazil

**Corresponding author**: Renato Sobral Monteiro-Junior (renato.monteiro@unimontes.br).

**Box I - 3-Point Subjective Effort Induction Scale – SEIS-3**

| **SCORE** | **EFFORT CATEGORY** |
| --- | --- |
| 1 | Weak |
| 2 | Moderate |
| 3 | Strong |

**Figure I – Residuals Partial Plots**


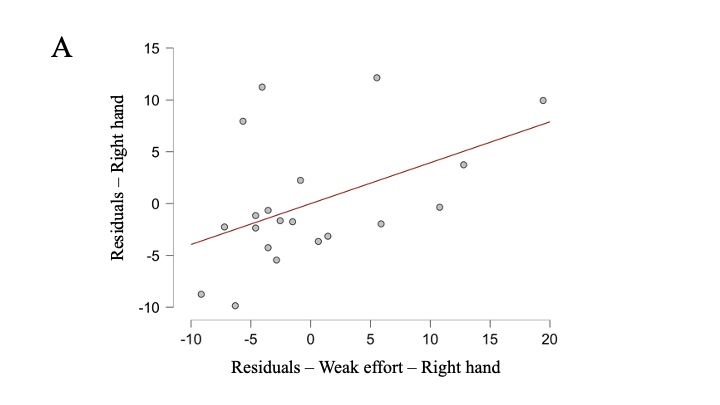


MIVC right hand versus weak effort of right hand.
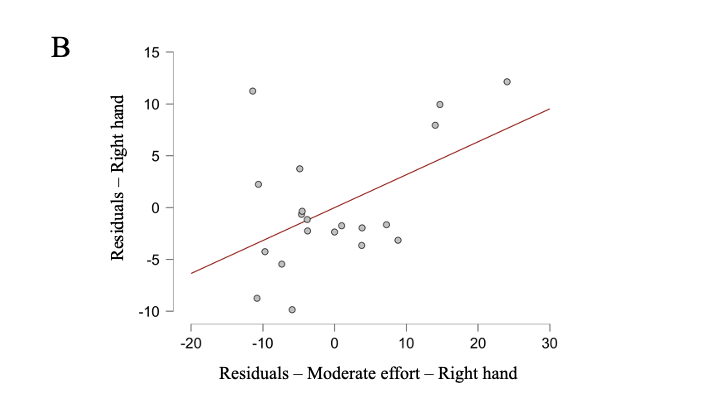


MIVC right hand versus moderate effort of right hand.


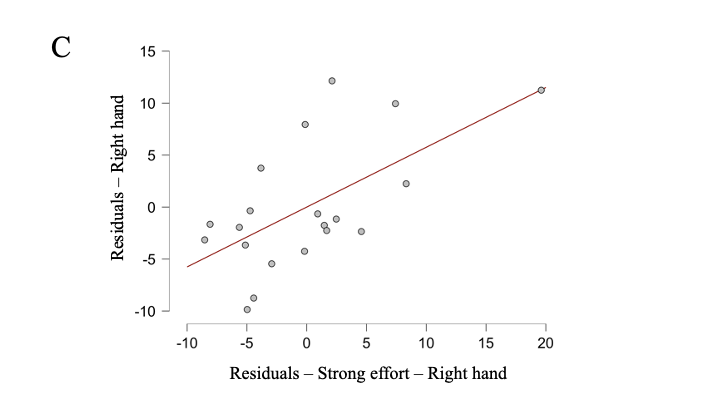


MIVC right hand versus strong effort of right hand.


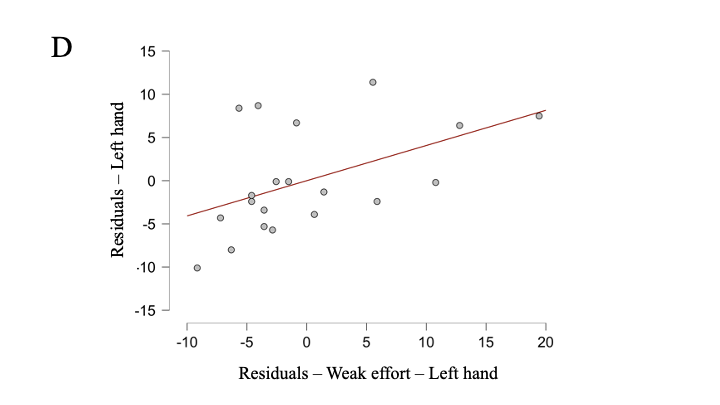


MIVC left hand versus weak effort of left hand.


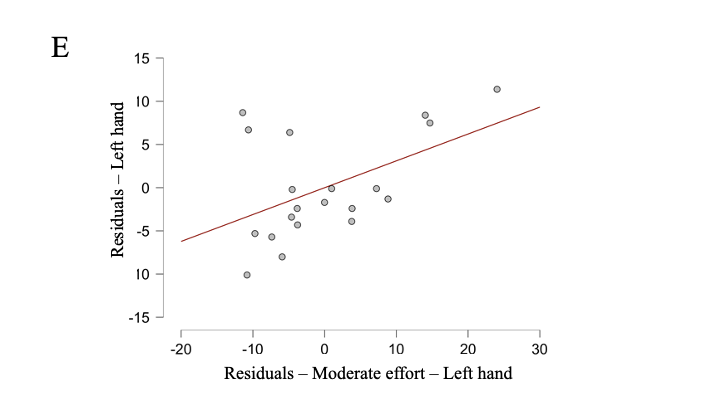


MIVC left hand versus moderate effort of left hand.


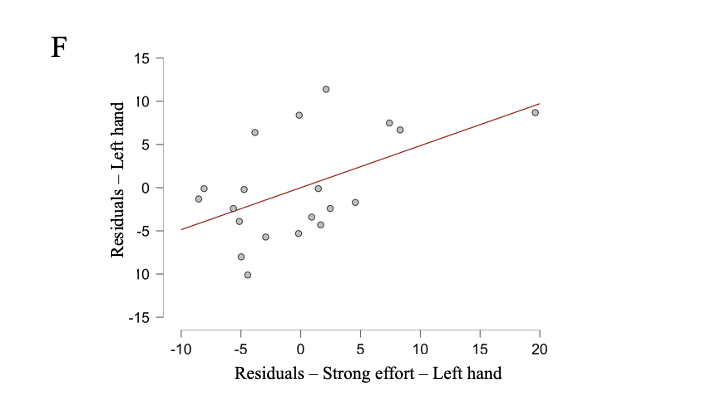


MIVC left hand versus strong effort of left hand.

**Figure II – Percentages (%) of maximal isometric voluntary contraction (MIVC) of each effort category of SEIS-3 with right (RH) and left (LH) hands.**


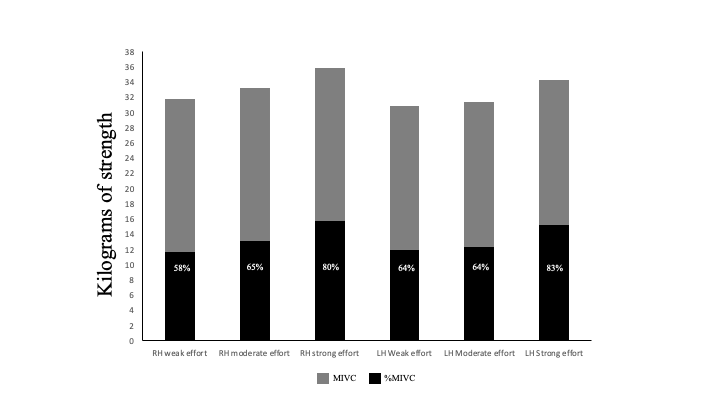

Supplement: Supplementary file 1 [file mmc1.docx]
